# Light and gravity signals synergize in modulating plant development

**DOI:** 10.3389/fpls.2014.00563

**Published:** 2014-10-28

**Authors:** Joshua P. Vandenbrink, John Z. Kiss, Raul Herranz, F. Javier Medina

**Affiliations:** ^1^Department of Biology, University of Mississippi, UniversityMS, USA; ^2^Centro de Investigaciones Biológicas (CSIC), MadridSpain

**Keywords:** gravitropism, phototropism, phytochromes, auxin, meristematic cells, cell cycle, ribosome biogenesis, space biology

## Abstract

Tropisms are growth-mediated plant movements that help plants to respond to changes in environmental stimuli. The availability of water and light, as well as the presence of a constant gravity vector, are all environmental stimuli that plants sense and respond to via directed growth movements (tropisms). The plant response to gravity (gravitropism) and the response to unidirectional light (phototropism) have long been shown to be interconnected growth phenomena. Here, we discuss the similarities in these two processes, as well as the known molecular mechanisms behind the tropistic responses. We also highlight research done in a microgravity environment in order to decouple two tropisms through experiments carried out in the absence of a significant unilateral gravity vector. In addition, alteration of gravity, especially the microgravity environment, and light irradiation produce important effects on meristematic cells, the undifferentiated, highly proliferating, totipotent cells which sustain plant development. Microgravity produces the disruption of meristematic competence, i.e., the decoupling of cell proliferation and cell growth, affecting the regulation of the cell cycle and ribosome biogenesis. Light irradiation, especially red light, mediated by phytochromes, has an activating effect on these processes. Phytohormones, particularly auxin, also are key mediators in these alterations. Upcoming experiments on the International Space Station will clarify some of the mechanisms and molecular players of the plant responses to these environmental signals involved in tropisms and the cell cycle.

## INTRODUCTION

Plants live in dynamic, ever-changing environments. To survive and thrive in these environments, plants have developed survival strategies to cope with the changing conditions (temperature, water, sunlight availability, etc.). Due to their stationary nature, plants have evolved growth-mediated movements that help them to adapt to changes in their surrounding environment. These directed growth movements (termed tropisms) help ensure the fitness and survival of the plant. For instance, plants generally direct root growth down into the soil (gravitropism) to help anchor the plant and absorb water (hydrotropism) and nutrients while directing shoot growth upward toward a source of light (phototropism; Molas and Kiss, 2009 and heliotropism; Vandenbrink et al., 2014). In addition, climbing plants such as vines send out tendrils that come into contact with an object and proceed to grow themselves around the object for support (thigmotropism).

The ability of plants to grow in response to environmental stimuli has been documented throughout history. Theophrastus, a disciple of Aristotle, noted the phototropic and heliotropic (modified phototropism) movements of plants. Theophrastus (erroneously) attributed the bending of a plant toward the sun as a byproduct of the sun’s rays removing liquid from the illuminated side of the plant (Theophrastus, 1976). The theory of water loss as the cause of phototropic growth was further championed by Bacon and Sylvarum (1627). In addition, poems dating back to ancient Rome detail observations of plants moving in response to the ever-changing position of the sun (Ovid, 2008). However, it was not until the publishing of Charles and Francis Darwin’s “*The Power of Movement in Plants*” that our current understanding of plant tropistic movements began to take shape (Darwin and Darwin, 1880). Darwin detailed experiments involving “heliotropic” movement (subsequently termed phototropic movement), plant circumnutation, responses to gravity as well as other nastic plant movements. In his experiments, Darwin detailed how plants sense external stimuli such as light and gravity and are able to respond through directional growth-mediated movements. In addition, Darwin outlined that perception of a stimulus and plant growth response do not necessarily happen in the same organ of the plant. Additionally, Darwin observed that the phototropic response was most distinct when the plant was illuminated with blue light, suggesting specificity in sensing the light source. His work also hypothesized the existence of a factor that moves from the site of stimulus perception to the sight of growth response, an idea that contributed to the discovery of the plant hormone auxin decades later.

Similar to phototropism, gravitropism has a long history of inquiry. One of the first characterizations of plant gravitropic response was detailed by Knight (1806). However, as noted in his paper, observations of gravitropic response long pre-dated his inquiry. To better understand the gravitropism in plants, Knight altered the perceived gravity vector by germinating garden beans on a wheel rotating perpendicular to the earth’s gravity vector. The beans germinating on the wheel responded to the new gravity vector by directing the growth of roots to the center of the wheel, and shoots toward its periphery, suggesting that gravity was responsible for plant orientation. In addition to Knight’s initial work, “*The Power of Movement in Plants*” was also seminal in the understanding of gravitropic movements. Darwin demonstrated that the tip of the root (root cap) was responsible for sensing the gravity vector through various dissection experiments. This observation led to the elucidation that once a new gravity vector was sensed, the root tip would produce a signal to promote differential cell growth on the two opposing sides of the root.

Darwin originally characterized the connection between gravitropism and phototropism in “*The Power of Movement in Plants*.” Since that time, efforts have been made to understand the relationship between the two processes, as well as trying to untangle the two to understand each in its own right. Since the initial characterization of phototropic and gravitropic movement, many of the underlying mechanisms of perception, transduction and response have been uncovered, yet little is known about the interplay between the two processes. Initially, studying the phototropic response alone was impossible as there was no way to remove the gravity vector on a terrestrial platform. Attempts to simulate the effects of reduced gravity via perpetual rotation on a clinostat or via free fall machines provide a proxy for microgravity by reducing the plant’s ability to perceive the gravity vector, but the gravity vector is never truly removed (Herranz et al., 2013a). In addition, these methods often have the unintended consequence of additional stresses being imparted on the sample. However, the advent of space research has allowed for the first true uncoupling of phototropic and gravitropic growth responses (Ferl et al., 2002; Wolverton and Kiss, 2009).

## GRAVITROPISM

**FIGURE 1:**
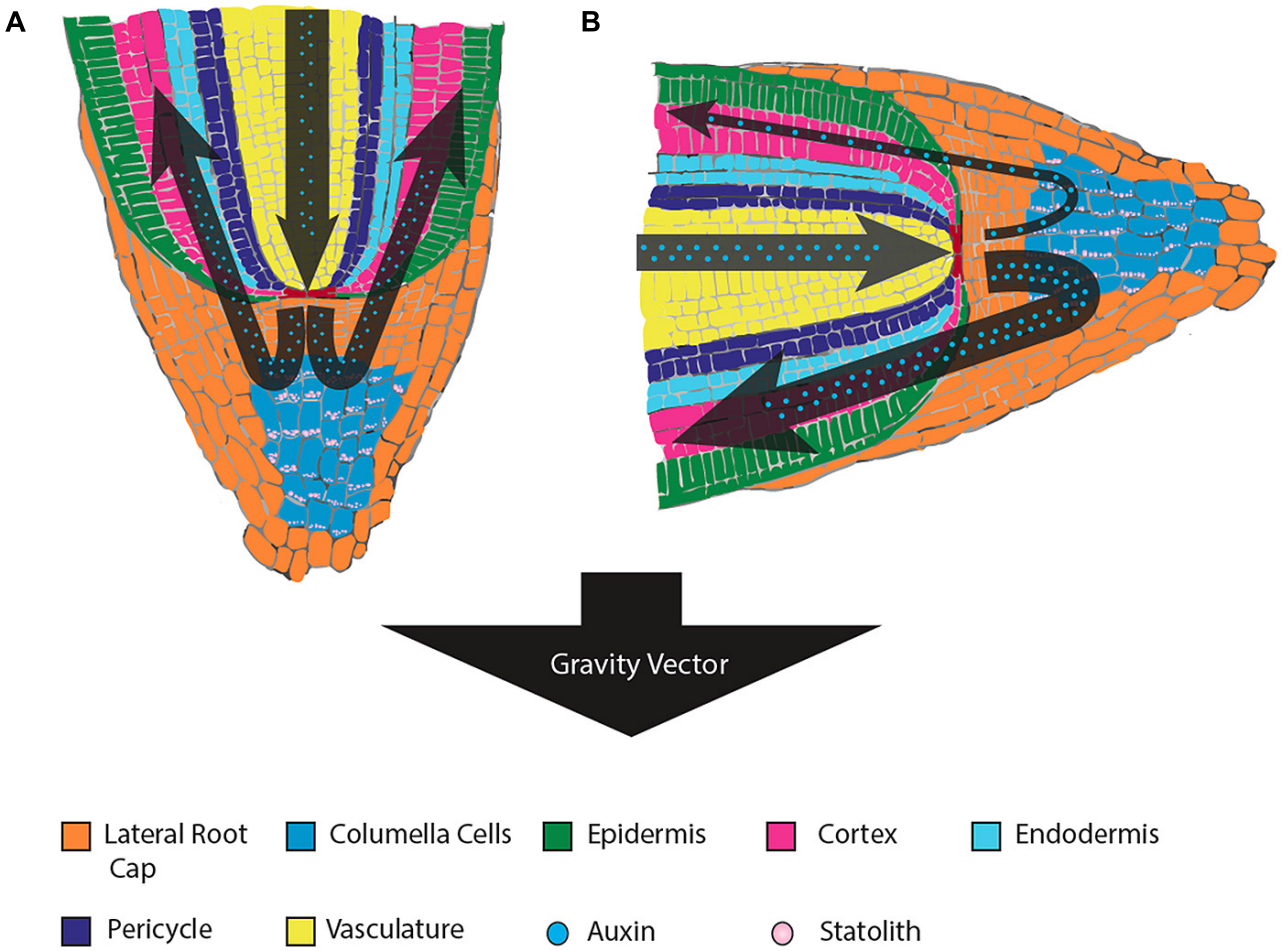
**Root tissue sensing and response to gravistimulation by reorientation. (A)** The statoliths of gravity-perceiving columella cells settle to the bottom of the cells relative to gravity, which results in a symmetrical distribution of auxin through all sides of the root cap and equal growth in the root elongation zone (apical of the root cap, not pictured). **(B)** Upon gravistimulation by reorientation, the statoliths settle at a new position toward the gravity vector. The new position is perceived by the cell, whereby a cascade of signals leads to an unequal distribution of auxin to the side of the root nearest the new gravity vector. This unequal distribution of auxin reduces cell growth where concentrations are high, resulting in growth-mediated bending of the root in the direction of the new gravity vector.

**FIGURE 2:**
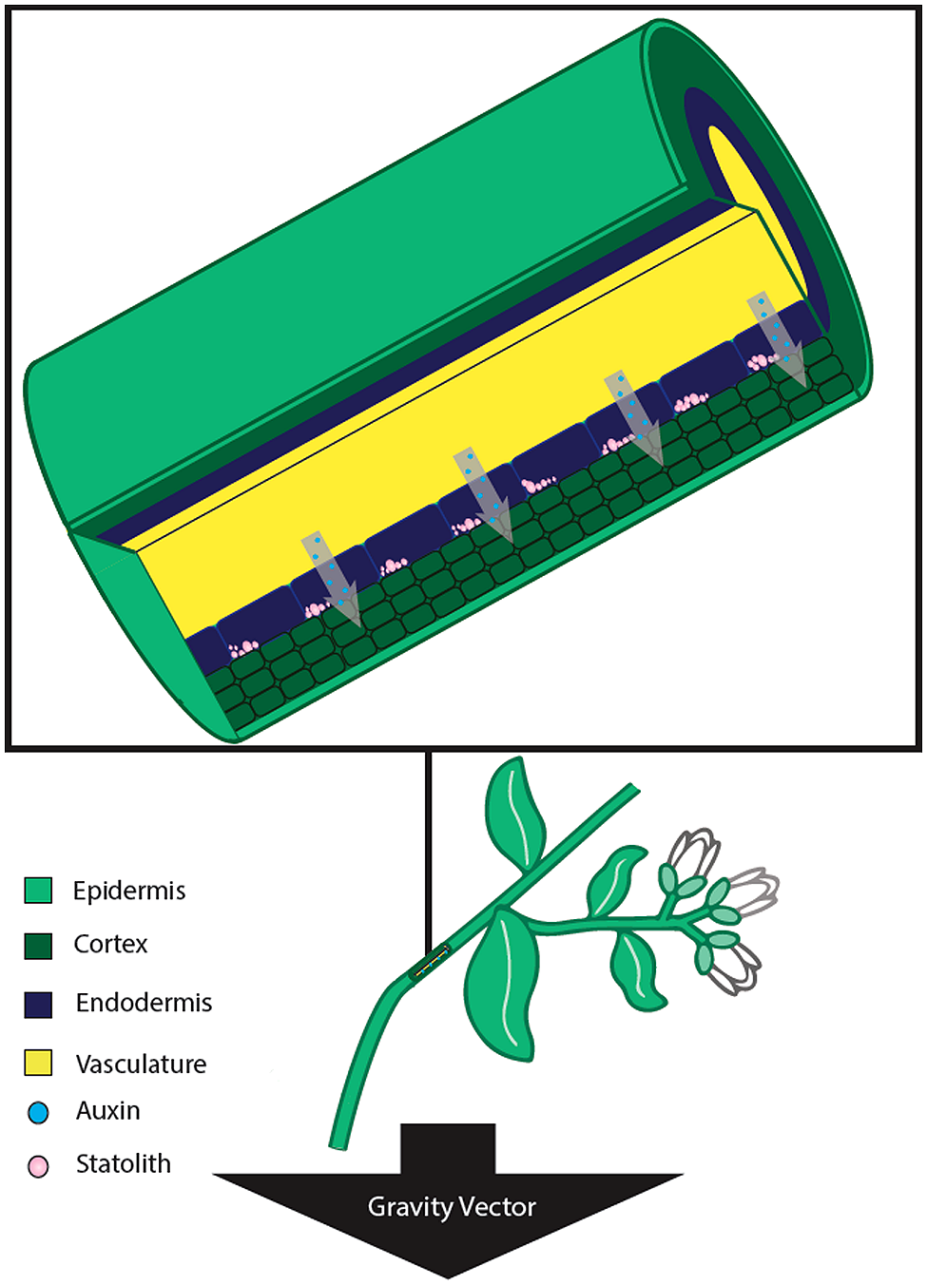
**Stem tissue sensing and response to gravistimulation by reorientation.** In stems, the gravity perceiving cells are located within the endodermis. Upon stimulation from a new gravity vector, the statoliths settle to the lateral side of the cell nearest the gravity vector. The new position of the statoliths leads to a differential increase in auxin concentration in the lateral tissue, and differential growth thereby occurs on the two opposite sides of the stem. This differential growth results in a bending of the stem away from the direction of the gravity vector.

While gravity is a constant vector that acts equally across all parts of an organism, higher plants contain specialized cells (termed statocytes) responsible for sensing gravity (Kiss, 2000; Saito et al., 2005). In flowering plants, the gravitropic response mechanism is localized primarily in two tissue types. In roots, specialized gravity sensing cells reside in the columella of the root cap (**Figure 1**), whereas evidence shows that plant shoots sense gravity via endodermal cells (**Figure 2**) – a single layer of cells between the vascular tissue and cortex (Fukaki et al., 1998; MacCleery and Kiss, 1999). These two specialized tissues are responsible for sensing the direction of the gravity vector and relaying the information to other areas of the plant for a response, such as differential growth along a zone of elongation (Sack, 1991). Generally light (phototropism) has an overriding effect on the gravitropic response of shoot tissue, however many studies have shown interplay between the two phenomena (Molas and Kiss, 2009).

### MECHANISM: SENSING OF GRAVITY AT THE ROOT AND SHOOT APICES

The starch-statolith hypothesis proposes that perception is mediated by the interaction of dense starch-filled organelles (termed statoliths) with other cytoplasmic structures in order to provide directional information to the plant (Kiss, 2000; Saito et al., 2005). A change in the position of the plant leads to a change in the potential energy of the amyloplasts, the statoliths in flowering plants. This energy is then transferred to the plasma membrane as the statoliths settle at a new position in the direction of the new gravity vector. This new position can then be relayed to the rest of the plant via the plant hormone auxin (Simmons et al., 1995; Firn et al., 2000) and then a gravitropic response can be initiated.

Roots most often display positive gravitropism, growing in the direction of the gravity vector and thus into the soil (**Figure 1**). When measuring the gravitropic response of roots of *Arabidopsis* starchless mutants, it was observed that the time of response was severely reduced and delayed compared to the response in wild-type genotypes. A similar response was observed in roots and hypocotyls of *Nicotiana* reduced starch mutants (Kiss and Sack, 1989, 1990). The gravitropic response of roots has also been shown to be linked to the actual rate at which amyloplast sedimentation occurs in *Arabidopsis* (MacCleery and Kiss, 1999). Furthermore, the gravitropic response has also been correlated to the total mass of statoliths in the root columella cells (Kiss et al., 1996, 1997).

In contrast to roots, stems and stem-like organs exhibit negative gravitropism, growing upward and away from the gravity vector. Mutants lacking amyloplasts in the endodermal cell layer lack gravitropic responses (Fujihira et al., 2000). In addition, stems of starch-deficient *Arabidopsis* correlated the total mass of starch in endodermal tissue to a change in the response to gravity (Kiss et al., 1997). In addition, gravity perception plays a role in plant development. Stem-like organs often maintain specific angles in relation to gravity, known as a gravitropic setpoint angle (GSA; Digby and Firn, 1995). The GSA operates through the existence of an antigravitropic offset mechanism that works in tension with gravitropism. The magnitude of the antigravitropic offset in relation to gravitropism determines the magnitude of the lateral stem’s angle. GSA values are modulated via the plant hormone auxin in the gravity sensing cells of root and shoot tissue, further implicating the role of auxin in tropism and plant architecture (Roychoudhry et al., 2013).

Further support for the starch-statolith hypotheses is provided by studies which reported that plants lacking starch in the stem endodermal amyloplasts also have a severely reduced gravitropic response (Weise and Kiss, 1999). This suggests that in stem the gravitropic response is similarly regulated by starch-filled amyloplasts. In addition, amyloplasts lacking a full complement of starch show reduced ability to perceive gravity in *Arabidopsis* (Kiss et al., 1996, 1997, 1998a,b). However, starch-deficient mutants lacking a full complement but grown in hypergravity environments (2–10 × *g*) restored the gravitropic response (Fitzelle and Kiss, 2001).

An alternative model to statolith-based gravity sensing is the protoplast-pressure hypothesis, where the total mass of the cytoplasm causes tension on the top and bottom of the plasma membrane (relative to the gravity vector; Wayne et al., 1990; Wayne and Staves, 1996). This model is largely based on studies of Characean algae *Nitellopsis* and *Chara*, both of which do not contain starch-filled amyloplasts yet still respond to gravity stimulus by exhibiting a gravity-dependent cytoplasmic streaming (Wayne et al., 1992; Staves, 1997). In addition, support for the protoplast-pressure hypothesis arises from the fact that starchless mutants of *Arabidopsis*, while greatly reduced, can still sense and respond to gravity (Kiss et al., 1989). Furthermore, proponents of the protoplast-pressure hypothesis point out that studies using starch mutants (key evidence in support of the starch-statolith hypothesis) do not discriminate effectively between the two competing hypothesis (Staves et al., 1997a). A study of gravitropism in the roots of rice (*Oryza sativa,* Poaceae) used variable densities of an external media to exert more or less force on root tissue without changing statolith sedimentation rate, resulting in changes in gravitropic response (Staves et al., 1997b).

While some debate still exists between the validity of the two models, it is likely a combination of multiple gravity sensing mechanisms that control gravitropism. It has been proposed that similar to the way in which a plant senses light, where multiple photosensory mechanisms exist, plants may contain multiple mechanisms which help to perceive the gravity vector (Sack, 1997). In addition, Perbal (1999) proposed that statoliths and the protoplast both can act in gravity sensing, with statoliths being the more sensitive mechanism. A commentary suggests that throughout evolution, higher plants have acquired multiple gravisensing mechanisms as evolution is unlikely to select against a process that aids in fitness (Barlow, 1995). Additionally, Barlow (1995) supposes that gravity sensory redundancy allows gravity to play a larger role in plant development through the evolution of distinct signaling pathways.

### TRANSDUCTION OF THE GRAVITY SIGNAL AND THE RESPONSE PHASE

Upon sensing of the gravity stimulus, the cell needs to convey the message to the elongation zone before differential growth leading to curvature can occur. In the case of stem tissue, the elongation region is nearby, requiring lateral transmission of the signal to this tissue (**Figure 2**). However, in the case of roots, the signal must be conveyed over a relatively longer distance to the root elongation zone (**Figure 1**). While various signal transduction mechanisms have been proposed, there is a great deal of evidence that supports the role of the plant cytoskeleton being involved in gravity transduction. It has been suggested that microtubules may be involved in the perception of gravity in coleoptiles (Blancaflor, 2002). In addition, depolymerization of the F-actin cytoskeleton results in promotion of gravitropic curvature in stems and roots, suggesting that the actin cytoskeleton may play a role in regulation of the gravitropic response (Yamamoto and Kiss, 2002; Hou et al., 2003).

While a role for the cytoskeleton in gravitropic response seems evident, little is known about the mechanism that associates the cytoskeleton with amyloplasts. The actin-tether model proposes that amyloplasts are physically associated with the cytoskeleton, and reorientation in respect to the gravity vector causes the amyloplasts to exert tension or slack on the cytoskeleton (Baluška and Hasenstein, 1997). This change in tension is then relayed to the plasma membrane, where the signal can then be transduced. A second hypothesis, the tensegrity model, proposes that amyloplasts are not attached to the cytoskeleton. Instead, as the amyloplasts reorient to a new gravity vector, they come into contact and disrupt the actin microfilament network. The signal is then transduced to the plasma membrane, where Ca^2+^ stores within the endoplasmic reticulum (ER) are released (Yoder et al., 2001).

As stated previously, the new relative position of the amyloplast (serving a statolith) is perceived by the cell. One observation is the statocyte responds to gravistimulation by increasing the cytosolic Ca^2+^ concentration and reducing the cytosolic proton concentration, which leads to auxin being differential distributed in the direction of the gravity vector (Tasaka et al., 1999; Morita and Tasaka, 2004). The resulting differential distribution of auxin results in unilateral inhibition of root growth on one side, causing the root to grow in a new downward orientation toward the gravity vector. It has been proposed that the ER acts as a Ca^2+^ reservoir; once statoliths settle in the direction of the gravity vector and contact the ER, stored Ca^2+^ is released into the cell (Perbal and Driss-Ecole, 2003). A study using high-resolution electron tomography adds support to this hypothesis, finding statolith sedimentation on the ER was sufficient in locally deforming the ER membrane, and in turn had the potential to activate local mechanosensitive ion channels (Leitz et al., 2009). The authors of this study further suggest that transmission of the gravisensory signal results from a combination of kinetic energy being transferred to the ER from the statolith, rapid release of kinetic energy from the ER upon initial reorientation of the columella cell, as well as statolith-driven motion of the cell cytosol. Furthermore, it is possible that calcium release acts as a signaling molecule during gravistimulation. A recent study indicates that an enzyme in the ethylene biosynthesis pathway (1-aminocyclopropane-1-carboxylate synthase) requires Ca^2+^ to elicit a gravitropic response, suggesting Ca^2+^ may be a signaling element for downstream growth regulation (Huang et al., 2013).

Perception of gravity by the root cap has been shown to result in asymmetrical auxin distribution across the root, leading to differential growth in the root elongation zone (Ottenschläger et al., 2003). A mathematical model inferring auxin distribution suggests a rapid (within 5 min) twofold increase in the auxin content on the lower side of the root, which leads to inhibition of growth and bending of the stem (Band et al., 2012). However, the mechanism and genes involved in the formation for the auxin gradient still remains unclear. Various genes (e.g., PIN, ABCB, and AUX/LAX families) have been implicated in the transport of auxin from the root cap to the distal elongation zone, yet a defined mechanism remains elusive (Luschnig et al., 1998; Petrášek and Friml, 2009). In addition to the identification of genes involved in auxin transport, regulators of the proposed transport genes such as the PIN regulator GOLVEN (GLV), which encode for small secretory peptides, have been shown to regulate the distribution of PIN2 and therefore the formation of the auxin gradient responsible for differential growth in gravitropism (Whitford et al., 2012).

A potentially important facilitator of auxin transport is *At*PIN3. PIN3 has been implicated in both hypocotyl and root tropisms. In the hypocotyl, PIN3 is expressed in the shoot endodermis, suggesting it may mediate the lateral distribution of auxin (Friml et al., 2002). In the columella cells of the root, PIN3 localizes along the plasma membrane symmetrically, but quickly (within ∼2 min) relocalizes to the bottom plasma membrane upon gravistimulation by reorientation. This relocalization of PIN3 coincides well with the redistribution of auxin along the root cap and endodermal cells to the elongation zone. This results in auxin inhibiting the growth of cells in the elongation zone nearest the gravity vector, causing the stem to grow downward toward the new gravity vector. Evidence shows that auxin efflux complexes such as PIN proteins cycle along the actin cytoskeleton between the plasma membrane and endosome (Geldner et al., 2001). These results are in accordance with models that suggest that the actin cytoskeleton reorganizes in concert with statolith sedimentation (Baluška and Hasenstein, 1997; Yoder et al., 2001). This reorganization would help to facilitate PIN3 relocalization to the new perceived bottom of the cell.

A recent study has shown that the regulation of PIN2 on opposing sides of gravistimulated root tissue is controlled by an auxin feedback mechanism (Baster et al., 2013). Auxin was shown to control the plasma membrane PIN abundance though vacuolar targeting and degradation through a specialized auxin receptor. This observation indicates that auxin plays a role in recycling the PIN proteins to the plasma membrane or to send them to the vacuole for degradation. Interestingly, the same study indicated that auxin leads to degradation of PIN in the presence of low auxin content as well. These results suggest that during a gravitropic response, auxin leads to the degradation of the PIN proteins coinciding with the different auxin gradients present during a gravitropic response. Additionally, a study by Löfke et al. (2013) has proposed that another plant hormone, gibberellic acid (GA), plays a key role in the trafficking of PIN proteins. Results showed that high GA promoted trafficking of PIN back to the plasma membrane, while low GA concentrations resulted in trafficking to the vacuole for degradation (Löfke et al., 2013). Taken together, these studies suggest that trafficking of PIN proteins is important in regulating auxin flow and initiating the differential growth response.

It is likely that AUXIN RESISTANT1 (AUX1) is responsible for the uptake of auxin in the gravity sensing tissue (endodermis and columella of the root cap), while PIN2 facilitates the transport from the root cap to the elongation zone (Friml, 2003). Genetic expression analysis of AUX1 suggests that AUX1 is required for root gravitropic response and is associated with basipetal auxin transport from the root cap to the elongation zone (Swarup et al., 2001). Recently, it has been proposed that AUX1/LAX genes are responsible for tissue specificity of high auxin levels, while PIN proteins are responsible for directing the flow of auxin from the root cap (Band et al., 2014). This evidence suggests that PIN and AUX/LAX proteins are key components of the signal transduction pathway from the columella cells to the elongation zone (Swarup et al., 2005). However, to date, many aspects of signal transduction between statocytes and the growth response remain unclear.

While sensing of the gravity vector is similar in both roots and shoots, it is possible that the mode of signal transduction is different. In roots, gravity perception takes place in the columella cells, and transferred to the elongation zone which is proximal to the columella cells (Kiss, 2000). Differential auxin concentrations between the two sides of the root cap have been correlated with bending of root tissue (Luschnig et al., 1998). In addition, while there is spatial separation of perception and response in roots, in shoots, perception and response occur in the same area (Molas and Kiss, 2009). It also has been suggested that perception of gravity occurs in the root columella cells and is transferred distantly to the shoot tissue, suggesting a larger role for root graviperception (Hopkins and Kiss, 2012).

Recent studies have also implicated plastid membrane proteins playing a key role in gravitropism. A mutation in *Arabidopsis* termed ARG1 (altered response to gravity) results in altered root and hypocotyl gravitropism without negative effects on other processes such as phototropism, root growth or starch accumulation (Sedbrook et al., 1999). The ARG1 gene encodes a DnaJ-like domain similar to cytoskeleton-interacting proteins. Additionally, studies have demonstrated that ARG1 is a membrane protein that potentially co-localizes with the PIN proteins (Boonsirichai et al., 2003). Furthermore, this study demonstrated that targeting of ARG1 to the endodermis or columella cells was sufficient in rescuing the phenotype in gravitropism-deficient mutants. ARG1 and its paralog ARL2 (ARG1-LIKE2) have been shown to associated in-complex with actin (Harrison and Masson, 2008a). Additionally, these two proteins are required for the asymmetrical distribution of auxin during gravitropic stimulation (Harrison and Masson, 2008b). ARG1 mutants have also shown to exhibit reduced plastid sedimentation, suggesting a role in both perception and signaling arising from gravitropic stimulation (Kumar et al., 2008). A study by Strohm et al. (2014) has shown that mutants of the Translocon at the Outer membrane of Chloroplast complex (TOC) show decreased gravitropic response in *arg1* mutants of *Arabidopsis*. This complex is responsible for the transport of nuclear-encoded proteins into plastids, however, the identity of which proteins need to be imported for a gravitropic response remains unclear. The study indicated that plastids not only play a role in gravity perception but also play a role in signal transduction. Another *Arabidopsis* mutant, *eal1* (endodermal-amyloplast less 1), completely lacks gravitropic movement in stems, while gravitropic response in roots remains unaffected (Fujihira et al., 2000). Map based cloning techniques have identified *eal1* as an allele of the transcription factor SHORT-ROOT (SHR; Morita et al., 2007).

Another group of compounds that have been implicated in gravitropic response is jasmonic acid (JA). JA increases significantly during gravitropic stimulation (Gutjahr et al., 2005). Not surprisingly JA has been shown to have multiple interactions with auxin (Riemann et al., 2003, and reviewed by Hofmann and Pollmann, 2008 and Wasternack and Hause, 2013). In rice coleoptiles, jasmonates have been shown to be present in a gradient opposite the auxin gradient during gravitropic stimulation (Gutjahr et al., 2005). In addition, lipozygenase, a key enzyme in JA-biosynthesis, has been shown to be up-regulated during gravitropic stimulation (León and Sánchez-Serrano, 1999). JA was induced by light as well as involved in photostimulation, where a rice mutant lacking JA biosynthesis (*hebiba*) exhibits delayed photodestruction of phytochrome A (Riemann et al., 2009). Furthermore, a study using *hebiba* identified the gene GDSL CONTAINING ENZME RICE 1 (*GER1*) as playing a role in gravitropic response, as it mirrors the level of JA in gravity stimulated coleoptiles (Riemann et al., 2007). The reduction in phytochrome A photodestruction results in an elevated growth response to red and far-red light. Jasmonates have also been implicated in growth-mediated response to touch (thigmotropism) in root tissue, suggesting a role for these compounds in multiple growth responses in plants (Falkenstein et al., 1991; Weiler et al., 1993; Stelmach et al., 1998; Blechert et al., 1999).

## PHOTOTROPISM

A large part of plant growth and orientation is directed through the tropisms. Initially when a seed is buried in soil, the seed relies on the gravitropism to direct its growth in the absence of light. Once the seedling emerges from the soil, phototropic processes can further direct the growth of the seedling. The phototropic response is most often observed in aerial tissues; however, phototropic responses do also occur in roots. Phototropism, which has been shown to be largely stimulated by blue light (Johnston, 1934; Kimura and Kagawa, 2006), further helps the plant to attain efficient growth through increased light capture. In response to blue light, plant shoots generally exhibit positive phototropic response while plant roots exhibit negative phototropic response (Sakai et al., 2000; Correll and Kiss, 2002). The phototropic response evolved early in plant evolution, being present in mosses and ferns in addition to angiosperms (Suetsugu and Wada, 2007). While mosses and ferns exhibit phototropic response to red light, phototropic growth in flowering plants is primarily a response to blue light.

### SENSING OF THE LIGHT SIGNAL

In flowering plants, phototropins are specialized photoreceptor proteins typically located on the plasma membrane that sense blue light and mediate a phototropic response (Sakamoto and Briggs, 2002). In addition to the blue light response, PHOT1 (Huala et al., 1997) and PHOT2 (Jarillo et al., 2001; Sakai et al., 2001) regulate stomatal opening, leaf expansion, and inhibition of stem growth among other processes (Christie, 2007). In the regulation of phototropism, PHOT1 is the primary photoreceptor, whereas PHOT2 only enacts a response when exposed to high intensities of radiation fluence (Sakai et al., 2001). While PHOT2 plays a small role in phototropic mediated signaling, this protein has significant roles in other light responses such as chloroplast movements (Wada et al., 2003) as well as stomatal regulation (Kinoshita et al., 2001). Upon low light conditions, PHOT2 stimulation causes relocation of chloroplasts perpendicular to incident irradiation, while high blue-light stimulation of PHOT2 leads to mobilization of chloroplasts away from the edge of the cell to avoid photobleaching of the organelles (Sakai et al., 2001).

Upon blue-light excitation, PHOT1 and PHOT2 release from the plasma membrane into the cytoplasm (Sakamoto and Briggs, 2002; Wan et al., 2008), while PHOT2 becomes associated with the Golgi apparatus (Sakamoto and Briggs, 2002; Kong et al., 2006). Following excitation with blue light, autophosphorylation of PHOT1 and PHOT2 leads to phototropin-mediated signaling (Inoue et al., 2008, 2011). Upon relocalization, PHOT1 has been shown to associate with clathrin, and is presumed to be internalized through clathrin-mediated endocytosis (Kaiserli et al., 2009). These studies indicate that phosphorylation and subsequent internalization of the PHOT1 is required for phototropic response, however the specific role PHOT1 and PHOT2 play in signal transduction remains unclear. Curiously, activation of phytochrome A, which is stimulated by red/far red light, prevents internalization of PHOT1 and leads to an increased phototropic response (Han et al., 2008).

In addition to the phototropins, there are multiple phototropin-interacting proteins shown to be involved in phototropism. Like the phototropins, NONPHOTOTROPIC HYPOCOTYL3 (NPH3) has been shown to localize to the plasma membrane and to physically interact with PHOT1 (Motchoulski and Liscum, 1999). In addition, NPH3 is required for phototropic response under both low and high intensity stimulation with blue light (Motchoulski and Liscum, 1999; Roberts et al., 2011). However, unlike the phototropins, NPH3 remains membrane associated throughout light stimulation and the phototropic response. It appears that NPH3 plays a role as a substrate in ubiquitination of PHOT1 under both low and high blue-light conditions (Roberts et al., 2011). Under high-blue-light conditions, when the plant is receiving sufficient light for photosynthetic processes, PHOT1 is poly-ubiquitinated and degraded. However, under low blue-light conditions, PHOT1 was shown to be only mono-ubiquitinated, a necessity for initiation of phototropic response (Roberts et al., 2011).

Another protein found to be involved in phototropism is ROOT PHOTOTROPISM2 (RPT2). RPT2 has been implicated in the phototropic response under high blue light conditions, where mutations in the gene result in defective hypocotyl phototropic response (Sakai et al., 2000). This is due in fact to RPT2 being transcriptionally regulated by both blue light and red light in a high-fluency-dependent manner (Sakai et al., 2000). Like the previously mentioned proteins, RTP2 also localizes to the plasma membrane of the cell, where it interacts with both PHOT1 and NPH3 (Inada et al., 2004) It is yet unknown whether or not RTP2 interacts with Cullin-3 (CUL3), however, Hohm et al. (2013) have suggested that both NPH3 and RTP2 could be required for ubiquitination under high blue-light condition.

### TRANSDUCTION OF THE LIGHT SIGNAL AND THE RESPONSE PHASE

Upon sensing a light signal, transduction of that signal must take place to induce growth in the elongation zones. The classical Cholodny–Went hypothesis proposes that the differential growth exhibited by a plant when illuminated with unidirectional light is a result of differential concentrations of auxin on the illuminated and shaded sides of the plant (Went and Thimann, 1937). The difference in auxin content results in cessation of cell growth on the illuminated side of the plant, while growth continues in the shaded side, resulting in tropistic movement in the direction of the light. A later study found that introduction of a physical barrier between illuminated and shaded sides of maize coleoptiles prevents the formation of an auxin gradient (Briggs, 1963). Using labeled auxin, Pickard and Thimann (1964) found that auxin moves laterally across the coleoptile when illuminated with continuous light, as well as low light dosages. This results in differential growth on the illuminated and shaded sides of the plant. The differential concentrations in auxin needed to spur differential growth and phototropic response are thought to be the result of local and long-range adaptations in auxin transport (Christie and Murphy, 2013; Hohm et al., 2013).

With the advancement of genetic and molecular techniques, it is possible to investigate the molecular mechanisms responsible for phototropic growth. Not surprisingly, similarities between phototropic stimulated signal transduction and gravitropic stimulated signal transduction have been identified. Like gravitropic response, PIN proteins play a significant role in auxin efflux during phototropic response. Five PIN family proteins (PIN1, PIN2, PIN3, PIN4, and PIN7) reside within the plasma membrane and appear to facilitate auxin efflux during phototropism, with PIN1 and PIN2 functioning as the main efflux carriers (Christie and Murphy, 2013). However, mutant screens of the PIN proteins suggest that all five contribute to phototropic response under differing conditions (Christie et al., 2011; Ding et al., 2011; Haga and Sakai, 2012). These proteins likely play an important role in formation of the lateral auxin gradient, however, it is likely that other auxin transporters exist to help facilitate in creation of the gradient (Christie et al., 2011).

Another family of proteins, ATP BINDING CASSETE B (ABCB) has been implicated in mediating phototropic response in *Arabidopsis*. Specifically, ABCB19 mutants have been shown to exhibit increased phototropic response (Noh et al., 2003; Kumar et al., 2011). It has been suggested that ABCB19 acts in concert with PIN1 to facilitate lateral transport of auxin through stabilization of PIN1 in the plasma membrane. Loss of function of ABCB19 results in reduced lateral auxin transport arising from the destabilization of PIN1, and the resulting loss of lateral auxin transport reduces the phototropic response.

In another similarity to gravitropic response, the AUX1 and LIKE-AUX1 (LAX) family of genes appears to play a role in influx of auxin and the resulting phototropic response. However, at the present time, the specific role of these proteins is unclear at best. Mutations to the AUX1/LAX genes results in very subtle loss of phototropic response when mutated alone (Okada and Shimura, 1992). However, when loss of function is introduced to AUX1 in combination with LAX2 and LAX3, reduced phototropic response in hypocotyl occurs (Christie et al., 2011).

## INTERACTION OF LIGHT AND GRAVITY: INSIGHTS OBTAINED FROM SPACEFLIGHT EXPERIMENTS

As evidenced by the commonalities between gravitropic and phototropic response, it is difficult to untangle the two processes without escaping the constant gravity vector present during terrestrial experiments. Once a phototropic response occurs in the plant, and differential growth occurs in response to the unidirectional light, a gravitropic response is initiated in reaction to the new perceived direction of the gravity vector (Okada and Shimura, 1992; Mullen et al., 2000). Special techniques such as rotation on a clinostat or free fall machines have been used in an attempt to simulate the effects of microgravity through constantly changing the gravity vector (Herranz et al., 2013a; Kiss, 2014). For instance, *Zea mays* grown on a rotating clinostat had reduced perceived gravity, which led to an increase in phototropic response (Nick and Schäfer, 1988). An alternative method to measure phototropic response is to use plants who lack the ability to sense the gravity vector. Multiple experiments conducted on mutant *Arabidopsis* plants lacking starch or amyloplasts displayed a greater magnitude of phototropic curvature (Vitha et al., 2000; Ruppel et al., 2001).

It was not until the advent of spaceflight that the effects of the gravity vector could be reduced and the decoupling of phototropism and gravitropism could be studied. Plants grown on the Space Shuttle or the International Space Station (ISS) provide the opportunity to observe phototropic response in conditions of microgravity. For instance, it has been revealed that in conditions of microgravity, amyloplasts do not distribute randomly throughout the statocyte, suggesting there is a connection between the amyloplasts and the cytoskeleton (Perbal et al., 1997; Smith et al., 1997; Driss-Ecole et al., 2000).

Experiments performed in low earth orbit aboard a Space Shuttle have been utilized to provide support for the starch-statolith hypothesis for gravity perception (Kiss et al., 1998b, 1999). Wild-type and starchless mutants grown in a microgravity environment, as well as in a centrifuge present aboard the shuttle that simulated 1*g* conditions, revealed that increased starch content increases the magnitude of gravitropic response. Studies on the ISS have been utilized to characterize a novel positive phototropic response to red light in *Arabidopsis* hypocotyls (Millar et al., 2010, Kiss et al., 2012). A similar phototropic response has previously been discovered in *Arabidopsis* roots by using specialized instrumentation on Earth (Kiss et al., 2003), however, conditions of microgravity revealed a much more robust response. These results suggest that flowering plants such as *Arabidopsis* have retained a red light phototropic response that is present in more ancient plant lineages such as ferns and mosses. However, it was also revealed that small fractional conditions (0.1–0.3 *g*) were enough to alternate the red-light phototropic response (Kiss et al., 2012). Interestingly, the phototropic response to blue-light stimulation also exhibits an exaggerated response in microgravity conditions (Millar et al., 2010).

In addition to signal perception, it is possible that microgravity environments affect the signal transduction of gravitropism as well. Evidence suggests that signaling via Ca^2+^ is hampered by microgravity environments (Ferl et al., 2002). Expression patterns of an *alcohol dehydrogenase::*β*-glucuronidase* (Adh::GUS) transgene could only be replicated when treated with Ca^2+^ inhibitors (Paul et al., 2001). It also has been found that other processes that utilize Ca^2+^ such as cell wall architecture, specifically lignin biosynthesis, is hampered during spaceflight (Cowles et al., 1994; Sato et al., 1999). Taken together, these studies suggest that signal transduction as well as perception are affected by conditions of microgravity.

While the microgravity conditions obtained during spaceflight offer a unique environment for understanding the interplay between gravitropism and phototropism, it also provides the ability to study plant growth and development in fractional or reduced gravity environments (Kiss, 2014). Gravity environments similar to those experienced on the moon or Mars can provide insight into the future plant growth that is required for long-range spaceflight and/or colonization of other celestial bodies. Continued experiments aboard the ISS (Kiss et al., 2014) will allow for a better understanding of the relationships between gravitropism and phototropism which will be experienced by plants grown by future colonization of other planets.

## GRAVITY AND LIGHT EFFECTS ON MERISTEMATIC CELL FUNCTIONS

### CELL PROLIFERATION AND GROWTH IN THE ROOT MERISTEM INFLUENCE PLANT DEVELOPMENT

All adult plants contain meristematic tissues, composed by populations of undifferentiated, totipotent cells with a high capacity of cell proliferation and cell growth. Any specialized tissue can be formed from meristems at any time in the life of the plant. Indeed, plant growth and development, which rely on cellular functions, greatly depend on the balance between cell proliferation and cell differentiation that exists in meristems, which is controlled, in turn, by hormones, among them auxin playing a central role (Perrot-Rechenmann, 2010).

Regulators of plant growth and differentiation (i.e., differentiation and developmental signals) are capable of activating key modulators of cell growth and cell division in a coordinated manner in meristems. Therefore, cell growth and cell proliferation are closely interconnected to one another and this coordination is called “meristematic competence” (Mizukami, 2001).

The concept of cell proliferation is intimately linked to the existence of the cell division cycle, or simply the cell cycle. Proliferating cells grow, duplicate DNA and divide in a cyclic manner. The process is strictly regulated at two specific checkpoints, the first of them at the transition G1/S, allowing DNA replication to proceed, and the second at the transition G2/M, in which the ability of cells to divide is checked. Specific proteins called cyclins, and specific cyclin-dependent kinases (CDKs) play essential roles in these regulatory processes (De Veylder et al., 2003). The concept of cell growth mostly affects the production of cell biomass, essentially proteins, so it is largely determined by the activity of RNA polymerase I, which controls ribosomal RNA synthesis and ribosome biogenesis (Baserga, 2007). It has been largely established, in particular for plants, that the rate of ribosome biogenesis can be estimated through certain features of the molecular cytology of the nucleolus (Medina et al., 2000; Shaw and Doonan, 2005; Sáez-Vásquez and Medina, 2008). Furthermore, regulation of ribosome production is controlled by a subset of nucleolar proteins, among which nucleolin is, a protein conserved in animals, plants and yeast, whose levels are correlated with the rate of functional activity of the nucleolus (Ginisty et al., 1999; González-Camacho and Medina, 2006; Pontvianne et al., 2007).

In this context, it is widely known that environmental conditions modulate meristematic activities, directly or indirectly, at different levels of regulation (Komaki and Sugimoto, 2012). Since many factors involved in light and gravity sensing are also acting in regulating cell cycle and ribosome biogenesis, it is interesting to review how these fundamental environmental factors affect plant development by affecting the regulation of these cellular processes.

### EFFECTS OF ALTERED GRAVITY ON CELL PROLIFERATION AND GROWTH

The influence of gravity on meristematic cell functions has been approached up to now in a relatively small number of experiments performed in space and in ground-based devices of simulated microgravity (Herranz et al., 2013a). In early pioneering studies on plant space biology it was reported an increase of mitotic index of lentil roots grown was found in microgravity (Darbelley et al., 1986). The interpretation was not unequivocal, since this effect could be due either to a shortening of the interphase or to a lengthening of mitosis. In a further study on board of Spacelab (IML-1 Mission), also using lentil seedlings, a lower mitotic index was shown in root cortical cells of samples grown in space, but no apparent perturbations in the mitosis were observed (Driss-Ecole et al., 1994). In the same work, the authors observed that microgravity promoted the arrest in the G2 phase of the cell cycle. All these experiments were performed with a relatively short exposure of plants to microgravity (28–29 h). In lentil seedlings grown for 30 h in microgravity, the progression of cell cycle was modified, even though the cell elongation did not appear affected (Legué et al., 1996; Yu et al., 1999). The densitometric analysis of nuclear DNA content of meristematic cells from roots grown in microgravity showed a decrease in the proportion of cells in S phase correlated with an increased proportion of cells in G1 phase, suggesting that the G1/S transition of the cell cycle is modulated by gravity.

The first European experiment on plant biology on board the ISS revealed that one of the most relevant effects of altered gravity is the disruption of the meristematic competence in cells of the root apical meristem (Matía et al., 2005, 2010). Under microgravity conditions, cell proliferation and cell growth appear uncoupled, losing their coordinated progress which is characteristic of these cells under normal ground gravity conditions.

Further experiments performed on ground-based facilities for microgravity simulation, including sequential sampling at different growth times and the analysis of gene expression, have confirmed the uncoupling of cell proliferation and ribosome biogenesis caused by altered gravity, showing that the weightlessness environment is a stress condition for plant proliferating cells. The effects of the gravitational stress are detected from the very beginning of germination, in 2-day-old seedlings (Matía et al., 2009; Herranz et al., 2013b; Manzano et al., 2013). The enhanced cell proliferation rate is not accompanied by an increase in the levels of cyclin B1, a regulator of the G2/M transition, as would be the normal in ground gravity, but, on the contrary, these levels appear depleted. At the same time, a lower cellular growth was observed, since ribosomes, the cellular factories of proteins, were produced at a lower rate. This depletion of ribosome biogenesis was already observed in meristematic root cells grown in simulated microgravity (Shen-Miller and Hinchman, 1995; Sobol et al., 2005, 2006), but it was not put in relation to other cellular processes. Since cyclin B1 is synthesized in the G2 phase of the cell cycle, and also this period is the most active in ribosome production (Sáez-Vásquez and Medina, 2008), a shortening of G2 phase is compatible with the mentioned observed uncoupling. The causes of this shortening could be found in a failure or malfunction of the cell size checkpoint which immediately precedes mitosis (De Schutter et al., 2007; González et al., 2007).

Whereas the effects of the lack of detection of a gravity vector on meristematic cells have been clearly identified, we still need to elucidate the factor triggering the cascade of functional events that eventually result in the alteration of meristematic cell proliferation and growth and in the disruption of meristematic competence. According to previously published data, the change in the hormonal signaling pathway mediated by the auxin polar transport could be a possible candidate to play this triggering role (Medina and Herranz, 2010).

**FIGURE 3:**
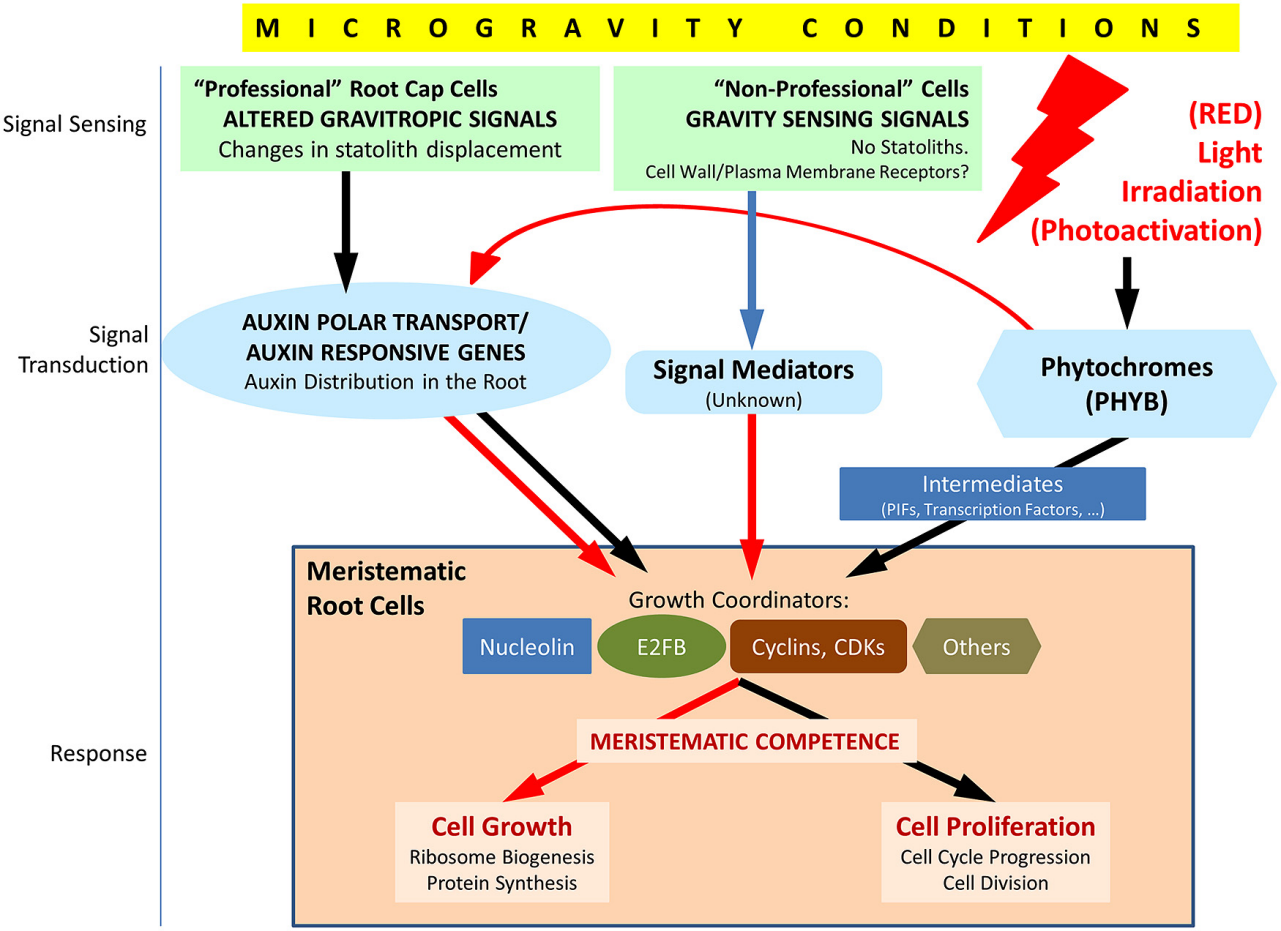
**Schematic view of the factors involved in the response of meristematic cells to the alteration of the environmental gravity sensing in conditions of microgravity and the possible counteracting effect of light irradiation, particularly red light.** Under normal gravity conditions, the gravity vector is sensed in statocytes of the root cap (“professional” cells), and the signal is transduced through the regulation of the auxin polar transport in the root. In meristematic cells, auxin regulates meristematic competence through its interaction with a number of growth coordinators appearing in the figure (nucleolin is a regulator of ribosome biogenesis which interacts, in turn, with cell cycle regulators; E2FB is a member of a family of transcription factors, regulated by auxin, which induces cell proliferation and growth; cyclins and cyclin-dependent kinases (CDKs) are major elements of cell cycle checkpoints controlling DNA replication and entry into mitosis). In addition, other mechanisms of gravity sensing and transduction must exist in “non-professional” cells, i.e., cells not specialized in gravity sensing, and, eventually, promote a similar response in meristematic/proliferating cells, as inferred from the results obtained in biological systems devoid of statocytes and statoliths. These mechanisms are poorly known at present. In microgravity conditions, auxin polar transport is inhibited, at least partially, auxin levels are high at meristems and growth coordinators are, in general, down regulated. The response is the disruption of meristematic competence in proliferating cells. Photoactivation by light (especially by red light), sensed and mediated by phytochromes (especially PHYB), is known to produce down regulation of auxin responsive genes and upregulation of many growth coordinators in meristematic cells, by means of direct and/or indirect mechanisms. These effects may counteract the gravitational stress in root meristematic cells in conditions of microgravity. Red arrows indicate down regulation, black arrows indicate up regulation and blue arrow indicates unknown effect.

It has been demonstrated that auxin plays a fundamental role in the connection between stimuli perceived by the plant and the cellular responses to them (Muday and Murphy, 2002). As stated above, mechanical sensing of a gravity change by columella cells is converted into a relocation of PIN proteins, finally resulting in changes in the auxin gradient in the root (Friml et al., 2002; Kleine-Vehn et al., 2010). There are experimental results showing that this change in the gradient is associated with the inhibition of the auxin polar transport, at least partially (Hoshino et al., 2007; Boucheron-Dubuisson et al., submitted). Auxin influences multiple aspects of plant growth and development, including the regulation of cell cycle progression and the coordination between cell growth and cell division (David et al., 2007; Jurado et al., 2010; Perrot-Rechenmann, 2010). Therefore, all available data point to auxin as the key mediator between altered gravity sensing and the observed effects on root meristematic cells (Medina and Herranz, 2010; **Figure 3**).

Since gravity can be considered as an abiotic component of the environment, it would be interesting to consider to what extent the response to gravity alteration follows a similar pattern as the response to other abiotic stresses, or, in the case of gravity, whether plants have developed specific mechanisms of defense and/or adaptation. It is widely known that abiotic stresses, such as thermal shock (heat or cold), drought or saline stress, usually cause arrest of the cell cycle on actively proliferating cells (Sacks et al., 1997; Burssens et al., 2000; West et al., 2004; De Veylder et al., 2007; Doerner, 2008; Komaki and Sugimoto, 2012). In the case of gravitational stress, the cycle is not arrested, but the regulation is severely affected through the failure of the G2/M checkpoint, which allows cells to enter mitosis before a critical size is reached. Apparently this scenario results in an increase of cell proliferation, but the daughter cells resulting from these divisions are abnormal, due to a reduced size.

Regarding the long-term consequences of these alterations, it could be foreseen that they could be dramatic for the plant survival, due to a serious impairment of plant development caused by the loss of meristematic competence. However, the reality is that plants finally are capable of surviving in space, even though some features of adult plants are not normal (Miyamoto et al., 1999; Link et al., 2003; Wolverton and Kiss, 2009). This observation suggests that plants would be capable of implementing some countermeasures against the gravitational stress, leading to some sort of adaptation or acclimation to the weightlessness environment, but the molecular processes and mechanisms involved in this presumed adaptation process are unknown at present. Indeed the genomic and transcriptomic data available on the specific cell cycle regulators whose expression is affected by gravity alteration are very scarce and limited to those scarce studies in which the analyzed material was enriched in proliferating cells, such as the root tip (Kimbrough et al., 2004). Therefore, the identification and discrimination of genes and proteins involved in cell proliferation and cell cycle playing a role in the response to gravitational stress is one of the most attractive challenges of space plant biology for the near future (**Figure 3**). Dedicated transcriptomic/proteomic studies using isolated meristems and/or proliferating *in vitro* cultured cells will be necessary to find accurate solutions to this problem.

### GRAVITY SENSING AND TRANSDUCTION IN “NON-PROFESSIONAL” CELLS

Whereas the role of auxin appears to be clear in connecting the alterations found in meristematic cells with the gravitropic response, it does not provide an explanation to the alterations found in cellular systems devoid of cells specialized in gravity sensing, such as *in vitro* cultured cells. A response to mechanical signals by these cells, in which the perception mechanism is unknown, has been reported in both plant and animal cells (Cogoli and Cogoli-Greuter, 1997; Kordyum, 1997; Dai et al., 2007). In plants, an altered gravity environment (including spaceflight) has been shown to induce transcriptomic and proteomic effects of on *Arabidopsis* callus cell cultures (Martzivanou et al., 2006; Barjaktarovic et al., 2009; Manzano et al., 2012; Paul et al., 2012; Hausmann et al., 2013). These cellular systems do not show any type of gravitropism, and cultured cells are not integrated in any organism possessing specialized mechanisms for gravity sensing. In this sense, cultured cells can be considered as “non-professional” cells (Van Loon, 2007). Regarding the effects of altered gravity on cell growth and proliferation parameters in this kind of cellular system, no specific reports have been published yet, but preliminary results indicate that the alterations are quite similar to those found on meristems (Herranz and Medina, 2014 and unpublished data).

Interestingly, whereas functional alterations on undifferentiated cells grown in microgravity were similar either in meristematic cells from seedlings or in cultured cells, transcriptomic changes were substantially different from seedlings or callus cultures (Paul et al., 2012). This suggests a specialized response by different cell types, such that both meristems and cell cultures would share the condition of being homogeneous populations of undifferentiated proliferating cells, whereas each seedling is composed by a heterogeneous collection of differentiated cells, differing in function, structure, and gene expression.

An intermediate case between gravitropic seedling roots and *in vitro* cell cultures is provided by seedling roots exposed to magnetic levitation (Manzano et al., 2013). In this case, root cells are subjected to the levitation force caused by the diamagnetic levitation of water, which counteracts the force due to gravity but it is not capable of inducing any displacement of statoliths. The response at the meristematic cell level is the disruption of meristematic competence associated to an altered polar auxin transport. This means that there should be an intermediate factor, independent of statoliths, capable of linking the signal sensed in the cell and the alteration of the polar auxin transport.

Therefore, depending on the type of cell, gravity sensing may or may not involve statolith movement and, consequently it may or may not produce gravitropic effects; furthermore, the transduction of the signal may or may not affect the polar auxin transport. In all cases, both *in planta* and *in vitro*, the response of undifferentiated proliferating cells to the perception of the lack of a gravity vector is an alteration in growth and proliferation capable of disrupting meristematic competence. It is conceivable that different mechanisms of gravity sensing and signal transduction (within a cell or throughout cells) involving different molecular and cellular players and mediators may exist in different biological systems and even co-exist in a biological model (plants or cell cultures; real or simulated microgravity; mechanical; or magnetic simulation). The nature and the localization of the mechanosensor in “non-professional” cells was hypothesized to be related to the membrane-cytoskeleton associations (Van Loon, 2007). There is a process, called gravity resistance, which is the capacity of plants to resist the gravity force by developing rigid structures, whose cellular basis is the existence of mechanosensitive ion channels at the plasma membrane and the reorientation of cortical microtubules (Hoson et al., 2005, 2010). This mechanism of gravity sensing is compatible with the available data on graviresponse in “non-professional” cells and may represent an initial explanation, which does not exclude the need of dedicated investigation (**Figure 3**). Furthermore, it has been proposed that cells outside the cap root area are capable of producing a partial gravitropic response in maize (Wolverton et al., 2002; Mancuso et al., 2006).

An alternative (or complementary) mechanism of gravity sensing, not directly related to columella cells, is the protoplast-pressure hypothesis, already mentioned and discussed in a preceding section of this paper.

### INFLUENCE OF LIGHT STIMULATION ON MERISTEMATIC CELL FUNCTIONS

#### Light signaling and plant development

From the different environmental factors which influence the life of plants on Earth, light via photosynthesis is ultimately the sole energy source for plant growth. Therefore, linking growth control to light signaling, at least during the most crucial developmental events, is essential for the most efficient use of energy and in order to guarantee success in plant growth and development. Consequently, light regulates many physiological processes related to plant development, starting by the earliest of them, namely seed germination and seedling morphogenesis (photomorphogenesis). This means that light plays specific roles in the activation and regulation of the cellular and molecular functions constituting the basis of these physiological processes, e.g., cell proliferation and cell growth.

Multiple parameters of the ambient light signal can be sensed by plants, such as light quantity (fluence), quality (wavelength), direction, and duration. The particular effect of the light direction and wavelength on establishing and modulating the growth direction of the plant and of the plant organs is the fundament of phototropism, which has been thoroughly analyzed in a previous section of this paper, in the presence and in the absence ofgravity.

It has been demonstrated that a significant portion of the genome shows differential expression between seedlings that are exposed to light and those that grow in darkness (Ma et al., 2001). The consequence is that many biochemical pathways, located throughout various subcellular organelles, in different cell types, are coordinately regulated by light. Thus, light ultimately controls the key mechanisms driving plant development, such as seed germination and seedling photomorphogenesis (Jiao et al., 2007). Seedling development is indeed quite different depending on whether it proceeds in the presence or in the absence of light. Seedling development in the dark is called skotomorphogenesis, or etiolation, whereas development under light is called photomorphogenesis, or de-etiolation. The morphology of etiolated and light-grown seedlings shows marked differences affecting the length of hypocotyls (shorter under photomorphogenesis), the size and morphology of cotyledons (smaller and closed under etiolation, with apical hooks) and the type of plastids and the presence of chlorophyll in them (chloroplasts versus etioplasts).

Furthermore, different metabolic pathways and cellular mechanisms show variable sensitivity to light signals of distinct qualities (Ma et al., 2001; Molas and Kiss, 2009). Phytochromes (PHY) constitute a family of proteins whose different members mediate the developmental responses to the different qualities of light, by exhibiting differential photosensory capabilities. In flowering plants, the *PHY* gene family consists of five members (*PHYA-E*; Molas and Kiss, 2009). From them, PHYA is the receptor of monochromatic far-red light, whereas responsiveness to monochromatic red light is predominantly attributed to PHYB (Tepperman et al., 2004).

Phytochromes are regulators of changes in gene expression induced in response to light sensing. The regulatory mechanism involves the activation of transcriptional networks, including a collection of transcription factors. Some of these transcription factors are regulated by just one type of light (wavelength), whereas many more respond to a wide spectrum of light (Jiao et al., 2007). A well-characterized, light-responsive element (LRE) is G-Box (Weisshaar et al., 1991; Hornitschek et al., 2012). The affinity of different factors for binding G-Box is modulated by post-translational mechanisms such as phosphorylation. The cases of G-BOX BINDING FACTOR 1 (GBF1) and COMMON PLANT REGULATORY FACTOR 2 (CPRF2) are representative of this mechanism. Both factors are phosphorylated by casein kinase II (CK2) to enable G-Box binding and promote the expression of photomorphogenesis genes in response to red light irradiation. In the case of CPRF2 phosphorylation, red light induces first its translocation to the nucleus, where it is then phosphorylated and bound to G-Box for promoting gene expression (Wellmer et al., 1999).

Other key elements driving the transcriptional change induced by light sensing are the members of a subfamily of basic helix-loop-helix (bHLH) phytochrome-interacting transcription factors, which have been designated PIFs (phytochrome interacting factors). PIFs selectively interact with the Pfr form of phytochromes, i.e., the photoactivated form already translocated to the cell nucleus. Different PIFs specifically interact with different phytochromes, and they define direct links between photoreceptors and transcriptional regulation. The analysis of several mutants of PIFs related to PHYB-mediated red light signaling shows phenotypes characterized by short hypocotyls and without any perturbation of light-induced expression of marker genes for chloroplast development. These observations indicate that these PIFs are negative regulators of PHYB signaling pathway (Huq and Quail, 2002) since they repress seedling photomorphogenesis in the dark (Leivar et al., 2008). Recent studies have revealed that PIFs are targeted for rapid degradation via the ubiquitin-proteasome pathway by photo-activated phytochromes in light (Leivar and Quail, 2011). A similar role can be attributed to PIFs interacting with other phytochromes.

Many genes have been identified as being regulated by PIFs. In turn, PIF activity is regulated by different pathways and factors, including various hormonal signals (reviewed by de Lucas and Prat, 2014). A recent study has provided evidence of the involvement of an evolutionarily conserved non-coding RNA in the modulation of red-light mediated photomorphogenesis by direct interaction with PIF3 gene transcription (Wang et al., 2014). Collectively, these results suggest that the PIF family functions as a cellular signaling hub in the phytochrome-mediated pathway controlling seedling photomorphogenesis (Leivar and Quail, 2011).

#### Light signaling is related to auxin signaling

Among the pathways regulated by phytochromes and PIFs, the interaction with hormone signaling, especially auxin, is of special interest. Indeed, multiple hormonal pathways are modulated by light to mediate the developmental changes and, conversely, hormone levels also serve as endogenous cues in influencing light responsiveness. In particular, a cross-talk has been shown to exist between PHYB-regulated responses to light signaling and hormone signaling. Mutants impaired in the synthesis or response to gibberellin (GA), brassinosteroids (BR) or auxin suppress the constitutive elongation phenotype of *phyB* mutants and cause de-etiolated growth in the dark (Alabadí et al., 2004; Nemhauser and Chory, 2004). The finding that PIFs play a direct role in the activation of auxin biosynthesis (Hornitschek et al., 2012) has provided a functional link between these different pathways and their integration to coordinate plant growth and development (**Figure 3**).

In addition, it is known that light regulates the expression of some *PIN* genes, encoding proteins responsible for the auxin efflux during the transport of this hormone. Enhanced auxin transport by these efflux carriers was reported to mediate the differential elongation of cells in the apical hook (Žádníková et al., 2010; Abbas et al., 2013) as well as during the shade avoidance response (Keuskamp et al., 2010).

Apart from PIFs, the transcription factor long hypocotyl 5 (HY5) is a convergence point of light and multiple hormone signaling pathways, such as GA, cytokinin, auxin and abscisic acid (Lau and Deng, 2010). Earlier studies have suggested that HY5 plays a role in suppressing auxin signaling (Cluis et al., 2004), a function which is performed through the activation of its negative regulators. A genome survey of the targets of HY5, using chromatin immunoprecipitation identified potential auxin signaling targets, including a dozen of auxin responsive factors (ARFs; Lee et al., 2007).

This relationship of light signaling with hormone signaling may have consequences in establishing the coordination of the growth of different plant organs. It is evident that changes in the environmental conditions do not affect the growth of all organs in the same manner, and, even within a given organ, not all cell types respond equally. Auxin is the most likely candidate to play the coordinator role, assuring balanced responses and equilibrated growth. The integration of the PIF regulatory network in these functions is crucial to drive the differential growth rates of the various plant organs during development and to provide plants with adaptation flexibility (de Lucas and Prat, 2014).

#### Light signaling controls cell cycle

One of the most striking characteristics of seedling photomorphogenesis is that the same light signal evokes largely different and sometimes opposite responses in different cells, tissues and organs. Thus, the response to light signaling is cell expansion in cotyledons, repression of growth in the hypocotyl and cell division and growth in meristems (Nemhauser, 2008). Regarding meristem activity, cell cycle progression in these cells is under the control of photoreceptors, as shown after monitoring a collection of mutants defective in phytochromes and cryptochromes (López-Juez et al., 2008). For this control, light initiates several hormonal responses associated with meristem function, among them auxin and cytokinin. A key role in this process is played by two central cell cycle regulators, the E2FB and E2FC transcription factors (Magyar et al., 2005; Jurado et al., 2007; **Figure 3**).

The investigation on the regulation of cell division by photoreceptors is less extensive compared to the research efforts devoted to other photomorphogenetic processes, such as cell elongation and greening. However, the most significant result that triggered the investigation on this topic was the observation that the growth of the shoot apex (meristem) is repressed in darkness but becomes rapidly activated by light (López-Juez et al., 2008). Light triggers the rapid downregulation of expression for specific transcription factors and genes, which leads to the loss of repressors that had been active in the dark. In this process, light also initiates rapid hormonal responses in the shoot apex.

Auxin levels are high in the shoot apical meristem in the dark. A large collection of auxin-responsive genes is highly expressed in the shoot apex in the dark and rapidly downregulated by light (**Figure 3**), indicating that increased auxin concentration and/or responsiveness could be part of the repressive mechanism of meristem function in the dark. The auxin-responsive transcription factor HAT4 was identified as an early red light-repressed gene in etiolated seedlings (Tepperman et al., 2004). On the contrary, the expression of the auxin transporter gene *PIN1* was shown to be upregulated by light in the shoot apex. However, these results could be due to direct the auxin flow away from the meristem upon light exposure (López-Juez et al., 2008).

At a later point after light activation, coinciding with leaf primordial development, a distinct cohort of auxin upregulated genes are expressed. This determines two stages of the auxin response to light: an early stage, associated with cell cycle activity, characterized by a drop of expression, and a later stage, associated with differentiation, characterized by elevation. Contrary to the results with auxin, cytokinin- and GA-responsive genes are activated by light (López-Juez et al., 2008).

During the light activation of meristem development, genes involved in ribosome biogenesis and protein translation are rapidly and synchronously induced, simultaneously with cell proliferation genes, such as B-type CDKs, some A-type cyclins and, slightly later, a group of D-, A-, and B-type cyclins (López-Juez et al., 2008; **Figure 3**). A similar program of gene expression was described for the process of activation of cell division of the root meristem that drives seed germination (Masubelele et al., 2005). The difference, as inferred from the particular genes, which are activated in each case, is that in root dormant seeds, cells are arrested in G1, whereas in the dark cells are arrested both at G1- and G2-phases. In any case, the synchronic expression of genes regulating cell growth and cell proliferation confirms that the coordination of these two processes (meristematic competence) is essential for the meristem function (Mizukami, 2001; Medina and Herranz, 2010).

In relation to the observed coordinated regulation of cell cycle genes, it was reported that light affected the levels of the E2F transcription factor family, constituted by transcriptional regulators playing important roles in the entry into cell proliferation or differentiation. Light increased the levels of E2FB (**Figure 3**) and decreased the levels of E2FC (López-Juez et al., 2008). E2FB is associated with the regulation of cell proliferation at the two main cell cycle regulatory events, namely the G1-to-S and the G2-to-mitosis checkpoints. The turnover of the E2FB factor is regulated by auxin, which induces the expression of the gene (Magyar et al., 2005). On the contrary, E2FC is a negative regulator of cell proliferation (Gutierrez, 2005). The effect of light on E2F transcription factors is dependent on DET (DEETIOLATED) and COP (CONSTITUTIVELY PHOTOMORPHOGENIC) factors, which are essential for the maintenance of skotomorphogenesis in the dark (Møller et al., 2002).

In addition to the role played in E2FB turnover, auxin mediates the light signaling effects on cell cycle by responding to changes in the red:far red light ratio. A reduction in this ratio causes arrest of cell cycle progression by inducing the expression of the *TIR1* (TRANSPORT INHIBITOR RESPONSE 1) gene, involved in auxin transport, and of *CKX6* (CITOKININ OXIDASE 6), which reduces the cytokinin levels (Carabelli et al., 2007).

Finally, an indirect pathway of cell cycle regulation by light, especially affecting the root meristem, is based on the depletion of sugars caused by the lack of light and the inhibition of photosynthesis (Nishihama and Kohchi, 2013). It was shown that the supply of glucose can reactivate the root meristem (Kircher and Schopfer, 2012). In fact, glucose was shown to activate the *TOR* (TARGET OF RAPAMYCIN) gene, which, in turn, activates E2FA transcription factor to promote expressions of S-phase genes (Xiong et al., 2013). Moreover, the nucleolar protein nucleolin, which activates ribosome biogenesis in connection to mechanisms of cell cycle regulation, is induced by glucose and sucrose (Kojima et al., 2007) Thus, the light signal perceived by leaves is transmitted by photosynthesis-derived sugars to the root meristem, where they activate cell cycle via different pathways, such as the TOR-E2FA.

#### Red light stimulates factors promoting meristematic competence

More than a decade before the literature reported in the preceding section on the light control of cell proliferation, a series of papers originated from the laboratory of Stanley J. Roux clearly demonstrated that light stimuli responsible for photomorphogenesis have activating effects on different factors promoting cell growth and proliferation and their mutual coordination, that is, what has been called meristematic competence.

Phytochrome-stimulating red light irradiation is capable of increasing the phosphorylation of nuclear proteins promoted by Ca^2+^ and calmodulin, an effect generally related to an increase in gene expression (Datta et al., 1985). Furthermore, it was shown that a red-light pulse given to dark-grown seedlings resulted in increasing the cell proliferation activity (i.e., the mitotic index) and in the expression of some relevant nucleolar proteins involved in ribosome biogenesis, and in particular, of a nucleolin-like nucleolar protein gene, indicating an increase in the rate of production of ribosomes in the nucleolus (Tong et al., 1997; Reichler et al., 2001; **Figure 3**).

## CONCLUDING REMARKS AND PERSPECTIVES

Disruption of meristematic competence appears to be a general effect produced by the lack of sensing of a defined gravity vector on undifferentiated proliferating cells of plants, regardless of whether they are meristematic cells from seedlings, or *in vitro* cultured cells. These results pose two important challenges. The first is the need of obtaining additional information on sensing, transduction and response mechanisms in the different model systems in order to establish the single or multiple pathways involved. The second is to search for countermeasures which could allow overcoming the gravitational stress on board space vehicles and in environments featuring gravity levels different from the Earth nominal levels.

With respect to the first challenge, the current extent of our knowledge is schematically summarized in **Figure 3**. Aspects of the details of these pathways remain unknown due to the lack of experimental data; however, these unknowns provide a summary of the research challenges on this topic in a near future. Thus, we will need to keep exploring altered gravity effects in cell cultures in order to uncover the details of mechanisms of the alteration of meristematic competence, and their potential dependence on auxin and/or on gravitropism-specialized organelles. In addition, further spaceflight missions are required to confirm these findings by using different mutants affecting auxin-responsive elements as well as cell proliferation and cell growth markers, e.g., nucleolin mutants.

Regarding the second challenge, the activating effect of light (particularly red illumination) can be of help in restoring meristematic competence under microgravity conditions (**Figure 3**). The factors which have been shown to be enhanced as a result of phytochrome-mediated photostimulation coincide with those which have been found to be decoupled under gravitational stress, producing the disruption of meristematic competence. In general, many points remain unknown in this cross-talk among different signals (light, gravity), mediators (auxin) and cellular effects (cell proliferation/cell cycle and cell growth/ribosome biogenesis). It is possible that the light stimulus, either alone or in combination with the alteration of gravity, could induce a redistribution of the auxin gradients in the root, which may be different from that produced by the alteration of gravity. The consequence at the cellular level could be a rate of cell proliferation and ribosome biogenesis which may reach similar levels as those existing on Earth.

Thus, in our upcoming experiments (termed the Seedling Growth Project) on the ISS, we aim to understand how gravity and light responses influence each other and to better understand the cellular signaling mechanisms involved in plant tropisms. Through these experiments, the red-light-dependent phototropic response in flowering plants will be further characterized. In addition, these experiments will consider the combined influence of light and gravity on plant development through the identification of changes in the mechanisms and regulation of root meristematic cell growth and proliferation. Auxin transport and perception will be analyzed as a regulatory process of these cellular functions, which also affects the developmental pattern of the plant. Experiments will test whether red light stimulation is capable of counteracting the effects of the gravitational stress on cell growth and proliferation. Finally, thresholds of response in fractional gravity will be measured to determine whether the red-light effect on blue-light-based phototropism is a direct or indirect effect. In the long term, experiments in fractional gravity produced by a centrifuge on the ISS will be performed to improve the understanding of how plants will grow and develop on the Moon and Mars.

## AUTHOR CONTRIBUTIONS

Joshua P. Vandenbrink, John Z. Kiss, Raul Herranz, and F. Javier Medina all had substantial contributions to the design of the work, have participated in the writing of the manuscript critically for intellectual content, approve the final version to be published, and agree to be accountable for the work.

## Conflict of Interest Statement

The authors declare that the research was conducted in the absence of any commercial or financial relationships that could be construed as a potential conflict of interest.
